# Role of Topical Nitroglycerin in Prevention and Management of Post-mastectomy Skin Flap Necrosis: A Prospective Randomized Controlled Study

**DOI:** 10.7759/cureus.115838

**Published:** 2026-09-05

**Authors:** Sumaiya Fathima Shabeer, Samibalaji Suppusamy, Vivekanand C., Srividhya Narayanasamy, Mohit Kumar Ramkumar, Vivek Harikumar, Dhanuksha Yadav, Sharath S Nair, Gokul S Nair, Neha V Damodar, Hariharan Ranganathan, Anandi Andappan

**Affiliations:** 1 General Surgery, Stanley Medical College and Hospital, Chennai, IND; 2 General Surgery, Government Peripheral Hospital, Chennai, IND; 3 General Surgery, Government Villupuram Medical College and Hospital, Villupuram, IND; 4 Oncology, Royal Shrewsbury Hospital, Shrewsbury, GBR; 5 General Medicine, Furness General Hospital, Barrow-in-Furness, GBR; 6 Emergency, Miracles Apollo Hospital, Gurugram, IND; 7 Internal Medicine, Max Healthcare, Gurugram, IND; 8 Surgery, King Edward Memorial Hospital and Seth Gordhandas Sunderdas (G.S.) Medical College, Mumbai, IND; 9 General Surgery, Government Tiruvannamalai Medical College and Hospital, Tiruvannamalai, IND

**Keywords:** breast, nitroglycerin, plastic, post-mastectomy, skin flap necrosis, surgery

## Abstract

Introduction: Mastectomy skin flap necrosis is a common postoperative complication following breast cancer surgery and is associated with delayed wound healing, increased morbidity, prolonged hospital stay, additional surgical procedures, and delayed initiation of adjuvant therapy. Impaired flap perfusion is considered the primary underlying mechanism. Topical nitroglycerin, through nitric oxide-mediated vasodilation, has been proposed as a simple and cost-effective method to improve flap vascularity and reduce ischemic complications. This study aimed to evaluate the efficacy and safety of topical nitroglycerin in preventing and reducing post-mastectomy skin flap necrosis.

Materials and methods: A prospective, randomized, assessor-blinded, controlled study was conducted on 60 patients undergoing modified radical mastectomy (MRM). Patients were randomly allocated into two groups of 30 each. The intervention group received topical 2% nitroglycerin ointment, while the control group received topical povidone-iodine ointment. The primary outcome was the incidence of skin flap necrosis. Secondary outcomes included wound healing time, delayed wound healing, surgical site infection, debridement requirement, hospital stay, severity of flap necrosis, and treatment-related adverse events.

Results: Skin flap necrosis occurred in two (6.7%) patients in the nitroglycerin group compared with 10 (33.3%) patients in the control group. Patients receiving nitroglycerin demonstrated significantly faster wound healing, lower rates of delayed wound healing, and reduced requirement for surgical debridement. Full-thickness flap necrosis occurred only in the control group, and the mean necrotic area was significantly smaller among patients treated with nitroglycerin. Although surgical site infection and hospital stay were lower in the nitroglycerin group, these differences were not statistically significant. Adverse events were mild and self-limiting, with no treatment discontinuations.

Conclusion: Topical nitroglycerin is a safe and effective adjunct for reducing post-mastectomy skin flap necrosis and improving wound healing outcomes. Its routine use may improve postoperative recovery and reduce flap-related complications following MRM.

## Introduction

Breast cancer is the leading cancer affecting women globally and continues to pose a substantial healthcare burden, particularly in low- and middle-income countries [1]. Modified radical mastectomy (MRM) remains one of the standard surgical treatments for patients with breast carcinoma, especially those presenting with locally advanced disease. Although surgical techniques have improved considerably, mastectomy skin flap necrosis continues to be a common postoperative complication, with reported incidence rates varying widely across studies [1]. This complication can result in wound breakdown, increased susceptibility to infection, prolonged hospitalization, the need for additional surgical procedures, unsatisfactory cosmetic outcomes, and delays in initiating adjuvant treatments such as chemotherapy or radiotherapy.

The development of skin flap necrosis is primarily attributed to inadequate microvascular blood supply after elevation of the mastectomy flap. Several interventions have been explored to enhance flap perfusion and minimize ischemic tissue damage. Among these, topical nitroglycerin has gained attention because it releases nitric oxide, producing local vasodilation that improves tissue perfusion and oxygen delivery [2]. Previous studies have suggested that topical nitroglycerin can decrease the incidence of mastectomy skin flap necrosis while promoting better wound healing, with minimal treatment-related adverse effects [2].

Evidence from recent systematic reviews and meta-analyses has further supported the potential role of topical nitroglycerin in reducing flap-related complications following breast surgery, although much of the available evidence is derived from retrospective and observational studies [3]. Owing to its affordability, ease of administration, and favorable safety profile, topical nitroglycerin has the potential to become a valuable adjunct in postoperative wound management. Therefore, the present study was undertaken to assess the effectiveness of topical nitroglycerin in preventing post-mastectomy skin flap necrosis and improving wound healing outcomes.

## Materials and methods

Study design and setting

A prospective, randomized, controlled, assessor-blinded study was conducted in 60 patients undergoing MRM at the Department of General Surgery, Stanley Medical College and Hospital, Chennai, Tamil Nadu, India, over a period of 12 months from November 2024 to October 2025. The study was approved by the Institutional Ethics Committee prior to commencement, and all procedures were performed in accordance with the ethical standards of the Declaration of Helsinki. Written informed consent was obtained from all participants before enrolment into the study. The study was registered with the Clinical Trials Registry of India (CTRI) under registration number 2024/04/065465 on April 9, 2024.

Study population

Female patients aged 18 years and above with histopathologically confirmed carcinoma of the breast who were planned for MRM were screened for eligibility. Patients with known hypersensitivity to nitroglycerin or nitrate-containing compounds, history of ischemic heart disease requiring nitrate therapy, severe hypotension (systolic blood pressure <90 mmHg), uncontrolled hypertension, chronic headache disorders, migraine, recurrent breast carcinoma, pre-existing skin involvement by tumor, ulcerated breast lesions, connective tissue disorders affecting wound healing, and those undergoing immediate breast reconstruction were excluded from the study. Patients who declined participation or were lost to follow-up were excluded from the final analysis, and the reasons for exclusion were documented according to the predefined study protocol.

Sample size and randomization

A total of 60 eligible patients were included in the study. The sample size was calculated based on an anticipated skin flap necrosis rate of 4% in the nitroglycerin group (Group A) and 30% in the control group (Group B), with a two-sided alpha level of 0.05 and 80% statistical power using a 1:1 allocation ratio. The calculated sample size was 56 participants (28 per group), which was rounded up to 60 participants (30 per group) to ensure an adequate sample size. Participants were randomly allocated in a 1:1 ratio into two groups comprising 30 patients each. Randomization was performed using a computer-generated random sequence prepared by an independent investigator not involved in patient recruitment or outcome assessment. Allocation concealment was ensured using sequentially numbered, opaque sealed envelopes that were opened only after completion of surgery. The outcome assessors remained blinded to treatment allocation throughout the study period, whereas treatment administration was performed by nursing personnel who were aware of the assigned intervention. Group A received topical nitroglycerin ointment 2%. Group B received topical povidone-iodine ointment as control treatment (Figure 1).

**Figure 1:**
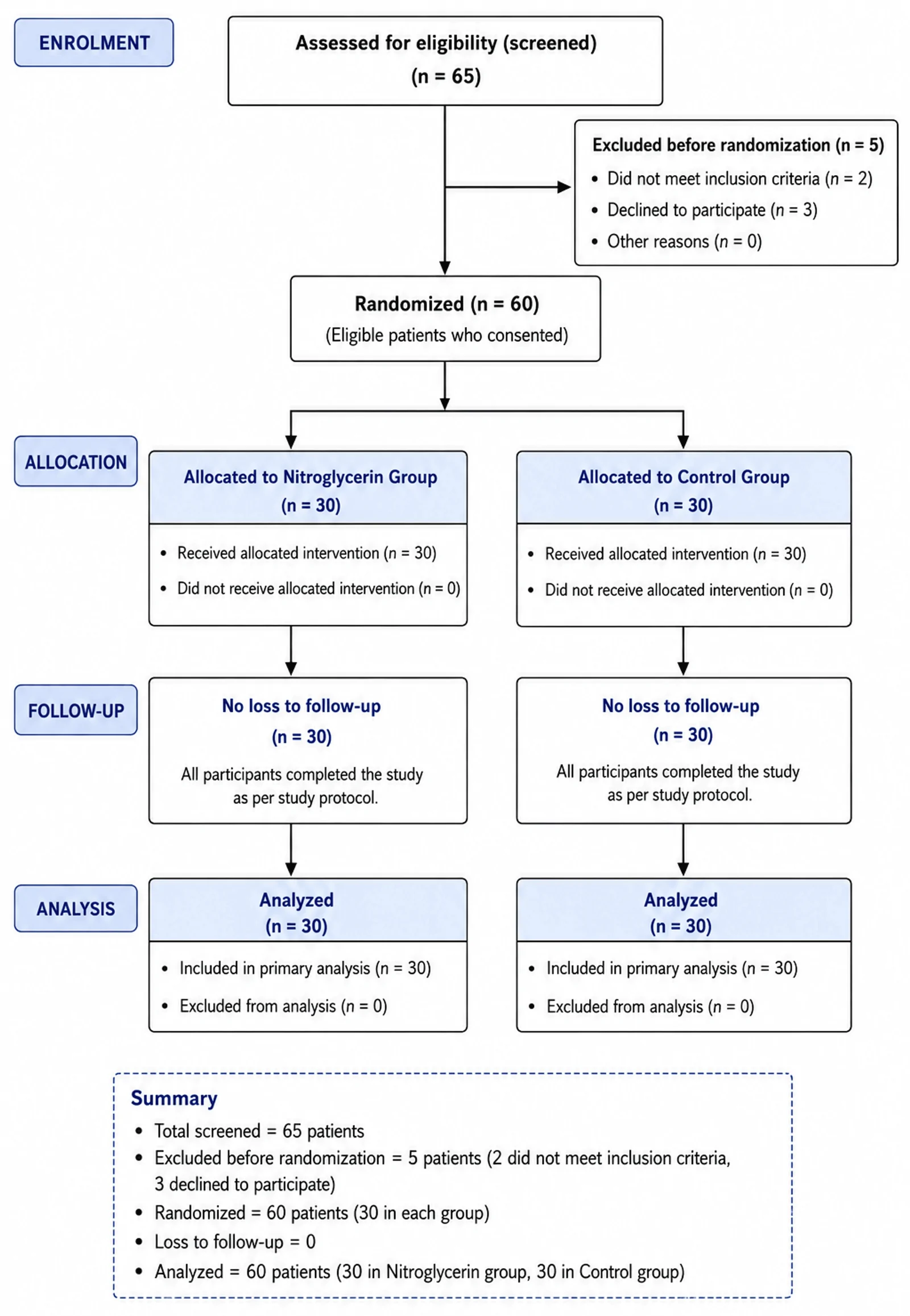
CONSORT flow diagram of this study CONSORT: Consolidated Standards of Reporting Trials

Preoperative assessment

Baseline demographic and clinical characteristics, including age, body mass index (BMI), menopausal status, comorbidities such as diabetes mellitus and hypertension, smoking history, tumor stage, and laterality of the disease, were recorded. Routine preoperative investigations, including complete blood count, renal function tests, liver function tests, coagulation profile, chest radiography, electrocardiography, and imaging studies, were performed as per institutional protocol.

Surgical procedure

All patients underwent standard MRM performed by experienced consultant surgeons using a uniform surgical technique. Immediate breast reconstruction using tissue expanders or implants was not performed in any participant, as patients undergoing immediate breast reconstruction were excluded from the study. Skin flaps were raised in the standard anatomical plane with preservation of an adequate and uniform flap thickness whenever oncologically feasible, while ensuring complete tumor clearance. Hemostasis was secured using electrocautery, and closed suction drains were placed in all patients. Postoperative care, including antibiotics, analgesics, drain management, wound dressing protocol, and discharge criteria, was standardized across both groups to minimize treatment bias.

Intervention protocol

The assigned topical treatment was initiated on postoperative day 2 after ensuring adequate hemostasis and flap viability. Postoperative day 2 was selected to allow confirmation of initial flap viability and adequate hemostasis while providing early intervention during the period when ischemic flap complications may become clinically apparent. In Group A, approximately 0.3 inch (7.5 mm) of 2% nitroglycerin ointment, equivalent to approximately 4.5 mg of nitroglycerin, was applied uniformly over the mastectomy skin flap while avoiding the incision line and drain exit sites. The ointment was applied three times daily for seven consecutive postoperative days. Patients in Group B received topical povidone-iodine ointment using the same application technique and frequency. Drug administration was performed by nursing personnel who were not involved in outcome assessment.

Outcome measures

The primary outcome measure was the incidence of mastectomy skin flap necrosis within four weeks after surgery. Mastectomy skin flap necrosis was defined as any area of partial-thickness or full-thickness skin loss resulting from compromised flap perfusion and characterized clinically by black discoloration, eschar formation, tissue sloughing, or devitalized tissue requiring prolonged wound care or surgical intervention [4]. Necrosis was categorized as superficial when limited to the epidermis and dermis and as full-thickness when extending into the subcutaneous tissue [4]. Secondary outcome measures included wound healing time, incidence of delayed wound healing, requirement for surgical debridement or secondary suturing, occurrence of surgical site infection, duration of hospital stay, and adverse events related to nitroglycerin therapy.

Wound healing time was defined as the interval between surgery and complete epithelialization of the wound without discharge, dressing requirement, or evidence of tissue necrosis. Delayed wound healing was defined as failure to achieve complete epithelialization within four weeks following surgery.

Surgical site infection was defined according to the Centers for Disease Control and Prevention (CDC) criteria and included the presence of purulent discharge, positive wound culture, localized signs of infection requiring antibiotic therapy, or infection diagnosed by the treating surgeon [5].

Adverse events monitored included headache, dizziness, flushing, hypotension, and local skin irritation. Hypotension was defined as a systolic blood pressure below 90 mmHg or a decrease of more than 20 mmHg from baseline requiring medical intervention.

Follow-up and outcome assessment

Patients were evaluated on postoperative days 2, 5, and 7 and subsequently at weekly intervals for four weeks. During each follow-up visit, flap color, temperature, capillary refill, evidence of necrosis, wound healing status, surgical site infection, drain-related complications, and treatment-related adverse effects were assessed and documented. Standardized digital photographs of the surgical site were obtained at each visit. Independent assessment of wound outcomes was performed by two consultant surgeons who were not involved in treatment administration and were unaware of treatment allocation; wound assessments were performed after removal of the postoperative dressing and any residual topical medication to minimize the possibility of identifying treatment allocation.

The collected data were entered into Microsoft Excel (Microsoft Corp., Redmond, USA) and subsequently analyzed using IBM SPSS Statistics, version 26.0 (IBM Corp., Armonk, USA). Continuous variables were summarized as mean ± standard deviation (SD), whereas categorical variables were presented as frequencies and percentages. The distribution of continuous variables was evaluated for normality using the Shapiro-Wilk test. Depending on the distribution of data, continuous variables were compared using either the independent samples t-test or the Mann-Whitney U test. For categorical variables, comparisons were performed using the Chi-square test or Fisher’s exact test, as appropriate. Relative risk (RR) reduction, along with its corresponding 95% confidence interval (CI), was calculated for the primary outcome wherever applicable. A p-value <0.05 was considered indicative of statistical significance for the primary outcome, while secondary outcomes were considered exploratory and were interpreted with appropriate caution without adjustment for multiple comparisons.

## Results

The baseline demographic and clinical characteristics were comparable between the nitroglycerin and control groups. No statistically significant differences were observed in any baseline variable (all p>0.05), indicating adequate randomization and homogeneity between the study groups prior to intervention (Table 1).

**Table 1:** Baseline demographic and clinical characteristics of the study population ^a^ Chi-square test; ^b^ Independent samples t-test. BMI: Body Mass Index; SD: Standard Deviation

Variable	Nitroglycerin Group (n=30)	Control Group (n=30)	Statistic	p-value
Age (years), Mean ± SD	52.8 ± 9.4	54.1 ± 10.1	0.51^b^	0.61
BMI (kg/m²), Mean ± SD	25.7 ± 3.2	26.1 ± 3.5	0.46^b^	0.65
Diabetes mellitus, n (%)	8 (26.7)	9 (30.0)	0.08^a^	0.78
Hypertension, n (%)	7 (23.3)	8 (26.7)	0.09^a^	0.77
Postmenopausal, n (%)	18 (60.0)	19 (63.3)	0.07^a^	0.79
Stage II disease, n (%)	21 (70.0)	20 (66.7)	0.08^a^	0.78
Right-sided mastectomy, n (%)	16 (53.3)	15 (50.0)	0.07^a^	0.80
Duration of surgery (min), Mean ± SD	118.6 ± 14.8	121.3 ± 15.2	0.69^b^	0.49

Skin flap necrosis occurred in two (6.7%) patients in the nitroglycerin group compared with 10 (33.3%) patients in the control group. Topical nitroglycerin was associated with an 80% RR reduction in flap necrosis (RR 0.20, 95% CI 0.05-0.84), and the difference was statistically significant (p=0.028) (Table 2).

**Table 2:** Comparison of primary outcome between study groups ^a^ Fisher’s exact test. RR: Relative Risk; CI: Confidence Interval

Outcome	Nitroglycerin Group (n=30)	Control Group (n=30)	RR (95% CI)	p-value^a^
Skin flap necrosis, n (%)	2 (6.7)	10 (33.3)	0.20 (0.05-0.84)	0.028

Patients receiving topical nitroglycerin demonstrated significantly faster wound healing and lower rates of delayed wound healing compared with controls. Although surgical site infection, duration of hospital stay, requirement for debridement, and secondary suturing were numerically lower in the nitroglycerin group, these differences did not reach statistical significance (Table 3).

**Table 3:** Comparison of secondary outcomes between study groups ^a^ Chi-square test; ^b^ Independent samples t-test. SD: Standard Deviation

Outcome	Nitroglycerin Group (n=30)	Control Group (n=30)	Statistic	p-value
Wound healing time (days), Mean ± SD	16.9 ± 3.4	21.2 ± 4.9	3.34^b^	0.002
Delayed wound healing (>4 weeks), n (%)	3 (10.0)	10 (33.3)	5.19^a^	0.023
Surgical site infection, n (%)	2 (6.7)	6 (20.0)	2.54^a^	0.111
Hospital stay (days), Mean ± SD	7.4 ± 1.6	8.5 ± 2.2	1.84^b^	0.071
Requirement for debridement, n (%)	1 (3.3)	6 (20.0)	4.04^b^	0.103
Requirement for secondary suturing, n (%)	1 (3.3)	4 (13.3)	2.05^a^	0.152

Although the incidence of superficial necrosis and full-thickness necrosis was numerically lower in the nitroglycerin group, the difference was not statistically significant. The mean necrotic area was significantly smaller among patients receiving nitroglycerin, indicating reduced severity of tissue loss (Table 4).

**Table 4:** Severity of mastectomy skin flap necrosis between study groups ^a^ Chi-square test; ^b^ Independent samples t-test; ^c^ Fisher's exact test. SD: Standard Deviation

Variable	Nitroglycerin Group (n=30)	Control Group (n=30)	Statistic	p-value
Superficial necrosis, n (%)	2 (6.7)	6 (20.0)	2.31^a^	0.128
Full-thickness necrosis, n (%)	0 (0.0)	4 (13.3)	4.29^c^	0.112
Mean necrotic area (cm²), Mean ± SD	1.2 ± 0.4	4.8 ± 1.6	3.92^b^	0.001

Topical nitroglycerin was generally well tolerated. Headache was the most frequently reported adverse event in the nitroglycerin group, although the difference did not reach statistical significance. No serious adverse events or treatment discontinuations were observed in either group (Table 5).

**Table 5:** Adverse events associated with topical treatment ^a^ Fisher’s exact test; ^b^ Chi-square test.

Adverse Event	Nitroglycerin Group (n=30)	Control Group (n=30)	Statistic	p-value
Headache, n (%)	5 (16.7)	1 (3.3)	2.96^b^	0.085
Dizziness, n (%)	2 (6.7)	1 (3.3)	0.35^a^	0.554
Flushing, n (%)	2 (6.7)	0 (0.0)	2.07^a^	0.150
Hypotension, n (%)	1 (3.3)	0 (0.0)	1.02^a^	0.312
Local skin irritation, n (%)	1 (3.3)	2 (6.7)	0.35^a^	0.554
Treatment discontinuation, n (%)	0 (0.0)	0 (0.0)	-	-

## Discussion

The present study showed that topical nitroglycerin significantly reduced the incidence of mastectomy skin flap necrosis compared with conventional wound care (6.7% vs. 33.3%). The observed effect size was consistent with previous evidence, with the present study demonstrating a crude odds ratio of approximately 0.14, compared with a pooled odds ratio of 0.17 (95% CI, 0.10-0.30) reported in a meta-analysis of randomized controlled trials [3]. Maintaining adequate skin flap viability is essential for optimal postoperative recovery because flap necrosis contributes to delayed wound healing, increased healthcare utilization, and postponement of adjuvant treatment. The favorable outcomes observed in our study are likely related to the ability of topical nitroglycerin to enhance local microvascular circulation through nitric oxide-mediated vasodilation, thereby improving tissue perfusion and limiting ischemic injury.

Our findings are consistent with the observations of Frey et al. [6], who objectively evaluated mastectomy flap thickness using magnetic resonance imaging and demonstrated that thinner postoperative flaps were significantly associated with ischemic complications. In their study, a postoperative flap thickness of less than 8 mm was an independent predictor of ischemic complications. Although flap thickness was not directly measured in the present study, our standardized surgical technique emphasized preservation of adequate flap thickness whenever oncologically feasible. The lower incidence of flap necrosis observed with topical nitroglycerin in our study therefore complements the evidence that both adequate flap thickness and tissue perfusion are important determinants of postoperative flap viability.

Likewise, Reintgen et al. [7] described flap ischemia as the principal pathological mechanism underlying mastectomy skin flap necrosis and demonstrated its association with increased wound morbidity and prolonged recovery. The reduced incidence of flap necrosis observed in patients treated with topical nitroglycerin in our study reinforces the importance of improving microcirculatory blood flow to minimize ischemic complications.

Previous clinical evidence has demonstrated a beneficial effect of topical nitroglycerin in reducing mastectomy skin flap necrosis. A systematic review and meta-analysis of randomized controlled trials by Wang et al. [3] reported a significant reduction in flap necrosis with topical nitroglycerin, with a pooled odds ratio of 0.17 (95% CI, 0.10-0.30). However, the timing and dosing regimens varied substantially among the included studies. In particular, the available evidence regarding initiation of topical nitroglycerin on postoperative day 2 is not uniform, with some experimental data failing to demonstrate a benefit when treatment was delayed until this time point [8]. In contrast, the present study observed a significant reduction in flap necrosis despite initiation on postoperative day 2. This discrepancy may reflect differences in study population, flap characteristics, dosing regimen, frequency of application, and clinical versus experimental conditions. Our findings therefore provide additional clinical evidence regarding the potential effectiveness of delayed postoperative initiation of topical nitroglycerin.

Similarly, Yao et al. [9] reported improved flap survival with topical nitroglycerin in patients undergoing breast reconstruction and highlighted its ease of use and favorable safety profile. Consistent with their findings, adverse effects in our study were infrequent, mild in severity, and did not necessitate discontinuation of treatment, supporting the practicality of incorporating topical nitroglycerin into routine postoperative care.

In addition to reducing flap necrosis, patients in the nitroglycerin group experienced significantly shorter wound healing times and a lower incidence of delayed wound healing. Enhanced tissue perfusion improves oxygen and nutrient delivery to healing tissues, thereby accelerating tissue repair. Although Mlodinow et al. [10] reported that flap-related complications adversely affect healing duration and postoperative recovery in patients undergoing tissue-expander reconstruction, their study population differs from that of the present study. Nevertheless, the association between flap-related complications and delayed recovery provides contextual support for our finding that reduced flap necrosis was accompanied by improved wound healing outcomes.

The requirement for surgical debridement was numerically lower among patients receiving topical nitroglycerin, although the difference was not statistically significant. This finding has important clinical implications because debridement increases patient morbidity, healthcare costs, and may delay subsequent oncological treatment. Similar conclusions were drawn by Vania et al. [11], who emphasized that preventing flap necrosis is the most effective strategy for minimizing additional surgical procedures and facilitating recovery. Our findings further strengthen this evidence.

Although the incidence of surgical site infection was lower in the nitroglycerin group, the difference did not reach statistical significance. Similar findings have been reported by Tang et al. [12], who suggested that postoperative wound infection is influenced by multiple patient- and procedure-related factors in addition to flap perfusion. Likewise, although patients receiving nitroglycerin had a shorter hospital stay, this difference may not have reached significance because of the relatively small sample size.

Another notable observation was the reduction in the severity of flap necrosis among patients treated with topical nitroglycerin. Full-thickness necrosis was observed only in the control group, although the difference was not statistically significant. However, the extent of tissue necrosis was significantly smaller in the intervention group. Robertson et al. [4] emphasized that the clinical impact of flap necrosis depends not only on its occurrence but also on its depth and extent. Likewise, Turin et al. [13] reported that extensive flap necrosis is associated with greater wound-related morbidity and poorer postoperative outcomes. The reduction in severe flap necrosis observed in our study therefore represents an important clinical advantage.

Topical nitroglycerin demonstrated a favorable safety profile throughout the study. Headache was the most frequently encountered adverse effect, whereas dizziness, flushing, and hypotension occurred only occasionally. No participant required discontinuation of treatment because of adverse events. These findings are consistent with recent clinical evidence demonstrating that topical nitroglycerin is generally well tolerated, with adverse effects typically being mild and transient [9].

Overall, the findings of the present study add to the growing evidence supporting topical nitroglycerin as an effective adjunct for improving mastectomy flap viability. Previous studies, such as that by Yao et al. [9], have consistently demonstrated its ability to reduce flap necrosis while maintaining an acceptable safety profile.

Limitations of the study

The study was conducted at a single tertiary care center with a relatively small sample size, which may limit the generalizability of the findings and reduce the power to detect differences in less frequent outcomes. Furthermore, the short follow-up period precluded assessment of long-term outcomes such as cosmetic results, scar quality, and patient satisfaction, while objective techniques for flap perfusion assessment were not employed. Although topical nitroglycerin may have practical advantages because of its availability and ease of administration, a formal cost-effectiveness analysis was not performed in the present study. Larger multicenter randomized trials with longer follow-up are needed to validate these findings and establish standardized protocols for the use of topical nitroglycerin following MRM.

## Conclusions

The findings of the present study indicate that topical nitroglycerin may serve as a safe and effective adjunct in both prevention and management of post-mastectomy skin flap necrosis. Compared with patients in the control group, those treated with topical nitroglycerin demonstrated a significantly lower occurrence of flap necrosis, more rapid wound healing, fewer instances of delayed healing, and a reduced need for surgical debridement. Furthermore, the extent of tissue injury appeared to be less severe among patients receiving nitroglycerin, as evidenced by a lower frequency of full-thickness necrosis and a smaller mean area of necrotic tissue. While a trend toward fewer surgical site infections and a shorter duration of hospitalization was observed in the nitroglycerin group, these differences were not statistically significant. The treatment was generally well tolerated, with adverse effects being mild, transient, and self-limiting, and none of the patients required discontinuation of therapy. Considering its simple method of application, favorable tolerability, and potential practical advantages, topical nitroglycerin could represent a practical adjunct for improving postoperative wound outcomes following MRM. Nevertheless, larger multicenter studies with standardized treatment regimens and longer follow-up are required to confirm these results and establish the optimal role of topical nitroglycerin in clinical practice.
